# Incidence of Hearing Loss in Preschoolers Presenting With Delayed Speech

**DOI:** 10.7759/cureus.75958

**Published:** 2024-12-18

**Authors:** Rashed Aldoseri, Isra Salem, Fajer Isa, Hamda Almansoori, Sara Almansoori, Noor Binsanad, Amal Faisal, Mai Nasser, Mohamed Alshehabi

**Affiliations:** 1 Ear Nose and Throat, Bahrain Defense Force Hospital, Royal Medical Services, Manama, BHR; 2 Ear Nose and Throat - Speech Therapy, Bahrain Defense Force Hospital, Royal Medical Services, Manama, BHR; 3 Ear Nose and Throat - Audiology, Bahrain Defense Force Hospital, Royal Medical Services, Manama, BHR; 4 Ear Nose and Throat, Bahrain Defense Force Hospital, Royal Medical Services, Riffa, BHR; 5 Consultant Otolaryngology - Head and Neck Surgery, Bahrain Defense Force Hospital, Royal Medical Services, Riffa, BHR; 6 Otolaryngology - Head and Neck Surgery, Bahrain Defense Force Hospital, Royal Medical Services, Riffa, BHR

**Keywords:** delayed speech, hearing loss, hearing test, sensory neural hearing loss, speech and language delay, speech delay

## Abstract

Objective: The aim of this study was to assess hearing level of preschoolers with delayed speech in order to detect any underlying hearing loss

Methods: In this research we targeted preschool children with speech delay, who have not been previously diagnosed with any medical or psychological illnesses. A total of 54 preschool speech-delayed children were audiologically assessed in our clinic in the past year. The age at time of referral ranged from two to 7.4 years. In this study, we focused on auditory brainstem response (ABR) test done to assess level of hearing.

Results: Out of the 54 children with delayed speech assessed in our clinic in the year 2023, there were 36 males and 18 females with a ratio of 2:1 male to female. Thirty-three children underwent the test and passed the test with normal hearing level. Only one child was found to have sensory neural hearing loss. A total of 20 (37%) children did not undergo the test as their parents noticed improvements in their speech lowering the possibility of hearing loss. A total of 23 patients have been diagnosed with neurodevelopmental disorders after referring them for psychological evaluation and IQ testing.

Conclusion: A great number of healthy preschool children with speech delay were found to have normal hearing. In this case, the otolaryngologist should be aware of the possible underlying clinical entities, especially of neurodevelopmental and psychiatric nature.

## Introduction

Speech is the physical act of verbal communication, while language is the system of symbols used for interpersonal interaction [1]. Speech delay occurs when a child fails to achieve expected language milestones within the typical age range. An infant starts engaging with their surroundings as early as the first few months of life, but recognizable words generally emerge between 12 to 15 months, following a predictable developmental pattern [2]. In 1994, the Joint Committee on Infant Hearing issued a position statement advocating for the universal detection of hearing loss in infants, ideally by the age of three months [3]. Many professionals in healthcare and special education have endorsed the early identification of hearing loss and subsequent intervention to enhance the language and academic outcomes for children who are deaf or hard of hearing [4]. Additionally, we have limited knowledge regarding the percentage of hearing-impaired children among those with speech and language delays, and we are almost completely unaware of the proportion of children with language delays who do not show other developmental deficits or anomalies [2].

## Materials and methods

This study investigated preschool children presenting with speech delays, specifically those who were medically stable and without identifiable syndromes, musculoskeletal anomalies, or any neurological or mental deficits. Due to the absence of other pathological indicators, these children were referred to our ENT department for comprehensive hearing evaluations by pediatric clinics, local health centers, and general practitioners. Over the course of one year, starting from January 2023 until December 2023, a total of 54 preschoolers with documented speech delays were assessed to evaluate their hearing capabilities. Initially, detailed medical histories were collected from the parents, which included inquiries about any familial occurrences of speech and language disorders. Following this, each child underwent a thorough otolaryngological examination. The presence of otitis media with effusion (OME) was identified as a reason to postpone audiological assessments, prompting the initiation of conservative treatment until resolution of the condition. The audiological evaluation encompassed tympanometry, free field testing, otoacoustic emission recordings, and auditory brainstem response (ABR) testing. This study primarily focused on the results from the ABR assessments to gauge the hearing levels of the participants. Based on the findings from the ABR testing, the children were categorized as follows: (a) normal ABR threshold, characterized by a clear and reproducible wave V recorded bilaterally at a threshold of 40 decibels normalized hearing level (dB nHL) within normal latencies; (b) mildly to moderately elevated ABR threshold, where wave V was elicited at stimulus levels ranging from 45 to 65 dB nHL; (c) significantly elevated ABR threshold, indicated by wave V produced at stimulus levels between 70 and 95 dB nHL; and (d) absence of ABR response, noted when no observable wave V was detected even at the maximum stimulus level of 95 dB nHL.

## Results

**Figure 1:**
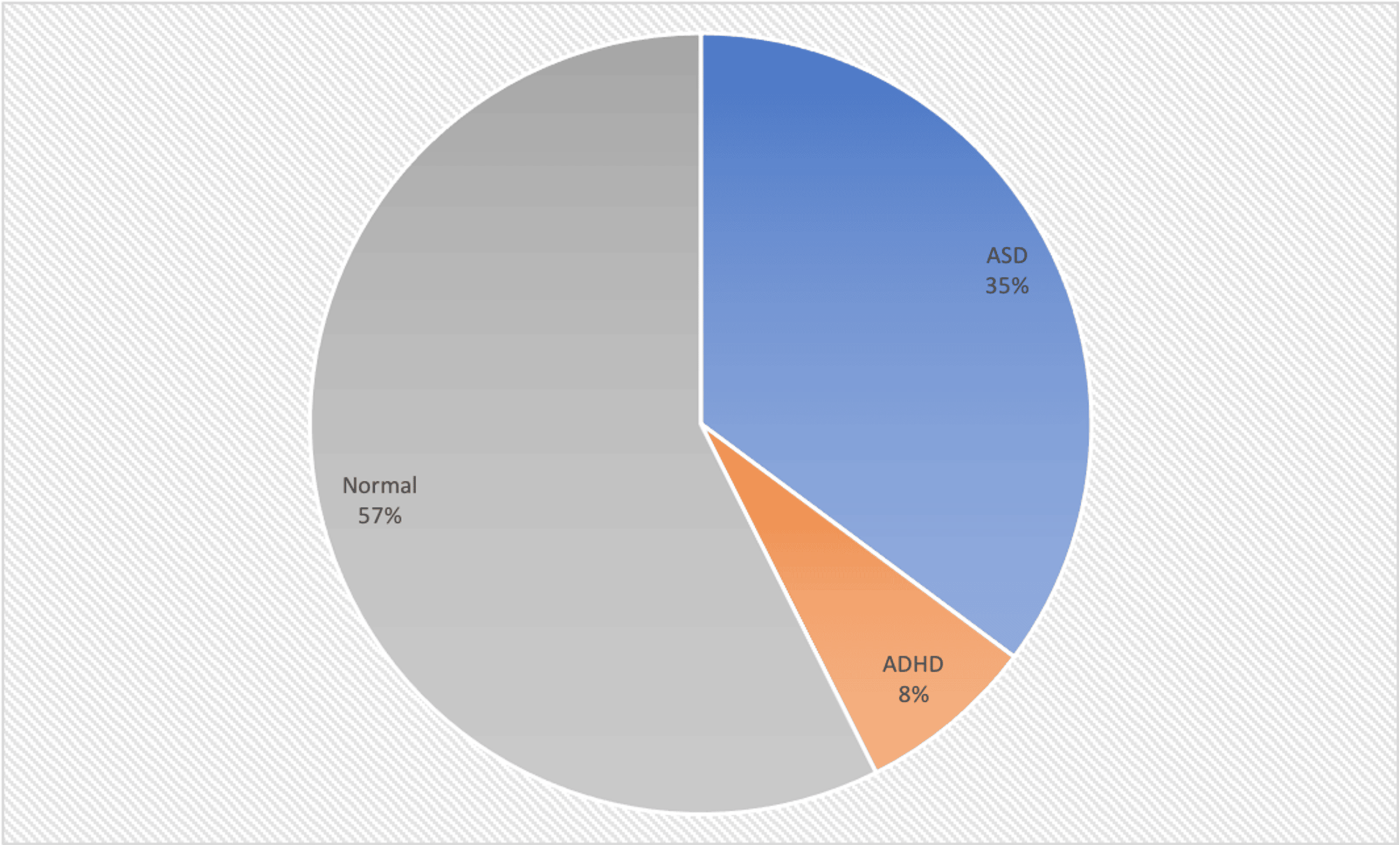
Psychological Evaluation Results ASD: autism spectrum disorder, ADHD: attention-deficit/hyperactivity disorder

In total, 54 patients were referred for hearing evaluations due to concerns of delayed speech development. The age of the children at the time of referral ranged from two to 7.4 years, with a median age of 3.9 years. This study exhibited a male-to-female ratio of 2:1 (Table 1). Out of the 54 patients, 34 patients (63%) completed the ABR evaluations; results indicated that their hearing levels fell within normal limits. Notably, only one patient (1.85%) was diagnosed with severe bilateral sensorineural hearing loss. The remaining 20 (37%) did not attend the audiological testing with ABR (Table 2). Follow-up assessments revealed that parents reported noticeable improvements in speech over time, suggesting a lower possibility of any potential hearing deficits in these cases. Importantly, no instances of conductive hearing loss were identified, likely attributed to the effective management of otitis media prior to the hearing assessment. In order to have a more comprehensive evaluation of the patients’ speech delay, we referred the patients for psychological evaluation and IQ testing. A total of 23 patients have been diagnosed with neurodevelopmental disorders with 19 (35%) diagnosed with autism spectrum disorder (ASD), four (8%) diagnosed with attention-deficit/hyperactivity disorder (ADHD). The remaining 31 (57%) patients had normal results (Figure 1). 

**Table 1:** Demographic Results

Demographics	
Total number of patients	54
Males	36 (66.6%)
Females	18 (33.3%)
Male to female ratio	2:1
Age range	2-7.4 years
Median age	3.9 years

**Table 2:** Auditory brainstem response (ABR) testing results SNHL: sensorineural hearing loss, CHL: conductive hearing loss

ABR Testing	
Normal Hearing	33 (97%)
SNHL	1 (3%)
CHL	0 (0%)

## Discussion

Delayed speech development has been a longstanding concern among pediatricians, primarily because it is often linked to a range of developmental challenges. This concern is well-founded, as delays in speech can have significant repercussions on a child’s social, emotional, academic, and vocational outcomes [3]. Early identification of speech delays and timely intervention can help minimize the cognitive, social, and emotional effects, leading to better long-term outcomes. Speech delays in children are typically viewed as arising from a combination of individual and multifactorial factors. Common contributors include myofunctional disorders, cleft palate, syndromic conditions, intellectual disabilities, psychological conditions such as selective mutism, receptive aphasia, cerebral palsy, and hearing loss. Interestingly, many children with speech delays appear physically healthy and show no other apparent anomalies or disabilities [5,6]. This presents a challenge for clinicians, who must differentiate between typical developmental variations and speech delays that could benefit from early intervention. A thorough evaluation, which includes medical, developmental, psychosocial, and family histories, is crucial for accurately assessing children with speech delays for appropriate management [7]. In our studied group, diagnosis of both ASD and ADHD have been made for a number of patients. While language impairment is not present in all individuals with autism, and there is no definitive prevalence estimate, there have been studies suggesting that approximately 50% of verbal children with autism experience structural language impairments [8]. The primary reason parents in many countries pursue a formal evaluation and diagnosis of ASD is concerns about delayed speech and language development or language skills that are below the typical level for their child's age group [8]. The American Academy of Pediatrics advises audiometric screening for children who meet any of the following criteria: failure to babble meaningfully by 12 months, no single words by 24 months, or fewer than 100 single words and no two-word phrases by 30 months. A speech delay is suspected if single words do not appear by 24 months or if two-word phrases are absent by 36 months [9].

The role of early and intact hearing in the development of language and speech is critical, as hearing loss during early childhood can lead to significant delays in speech and language skills. Hearing impairment can be classified as either conductive or sensorineural. Conductive hearing loss is commonly associated with conditions like otitis media with effusion, middle ear malformations, or atresia of the external auditory canal. Sensorineural hearing loss, however, may be caused by factors such as intrauterine infections, kernicterus, ototoxic drugs, bacterial meningitis, hypoxia, or intracranial hemorrhage [10]. The findings in this study were largely based on ABR testing, which, despite its limitations, remains a useful tool for assessing hearing status, especially when behavioral testing is not feasible or yields inconclusive results. ABR findings are strongly correlated with behavioral pure-tone thresholds, particularly in the 2-4 kHz range, which is critical for speech perception. Therefore, ABR audiometry provides reliable data for hearing assessment, as evidenced by our study, where the majority of participants had normal hearing [2]. Conductive hearing loss, often resulting from acute otitis media or otitis media with effusion, is the leading cause of intermittent, mild-to-moderate hearing loss in infants and young children [11]. In contrast, acquired sensorineural hearing loss (SNHL) is most commonly associated with bacterial meningitis. Autosomal dominant, non-syndromic SNHL is frequently identified during routine school-age audiological screenings [12]. Certain forms of syndromic SNHL are also diagnosed at this age, usually when associated comorbidities, previously undetected during physical exams, come to light [13]. Research consistently supports the benefits of early intervention for children with hearing loss, such as the use of hearing aids, which has been shown to significantly improve both social and academic outcomes, including speech development [14].

One limitation of this study is its small sample size, as it only includes patients who were seen within the past year. Patients from earlier years were excluded because ABR testing was temporarily suspended during the COVID-19 pandemic. Additionally, our institution has historically employed a variety of diagnostic approaches beyond ABR testing.

## Conclusions

This study explores the speech delay in pre-schoolers, emphasizing its significance in early childhood development. It is important for physicians to explore all possibilities in patients presenting with speech delay as early intervention is essential for preventing potential setbacks in cognitive development. Without timely treatment, a child's ability to communicate effectively may be compromised, hindering their overall growth. This paper identifies various factors contributing to speech delay, with a particular focus on hearing loss. Additionally, it provides an explanation of hearing assessments, specifically detailing the ABR test, which was utilized in our research.
